# Angioid streaks - a rare cause of neovascular glaucoma. Case report.

**Published:** 2014

**Authors:** E Ungureanu, A Geamanu, I Careba, M Grecescu, S Gradinaru

**Affiliations:** * University of Medicine and Pharmacy "Carol Davila"; ** University Emergency Hospital Bucharest, Romania – Department of Ophtalmology

**Keywords:** neovascular glaucoma, Bevacizumab, angioid streaks

## Abstract

**Rationale.** Neovascular glaucoma is the type of glaucoma most refractory to treatment. The most frequent causes are those associated with retinal hypoxia, such as proliferative diabetic retinopathy, central retinal vein occlusion, branch retinal vein occlusion, central retinal arterial occlusion, ischemic ocular syndrome etc.

Rare causes of neovascular glaucoma are multiple and are due to VEGF synthesis associated with chorioretinal inflammations or degenerations.

We present a case with neovascular glaucoma associated with an extremely rare cause, angioid streaks

**Objective.** The objective of our prsentation was to asses efficacy of the 5-FU associated trabeculectomy following bevacizumab intravitreal administration

**Methods and results.** Case report of a 48 years old female patient which presented at the emergency room with painful red left eye. At presentation best corrected left eye visual acuity was 1/10, intraocular pressure was 36 mm Hg. Examination established the diagnosis of Neovascular glaucoma associated with angioid streaks. After intravenous Manitol, oral Acetazolamide and topical treatment with fixed combination timolol-brinzolamide, topical steroid and mydriatic intraocular pressure decreased. Intravitreal bevacizumab injection was performed, followed after 3 weeks by trabeculectomy.

**Discussion.** Angioid streaks are an extremely rare cause of neovascular glaucoma. The treatment is similar to the treatment for other causes of neovascular glaucoma

## Introduction

Neovascular glaucoma is one of the most complex types of glaucoma. The mechanism is aqueous drainage impairment due to a fibrovascular membrane which covers anterior chamber angle (the secondary open angle stage) or due to the closing of the angle following fibrovascular membrane constriction (the secondary closed angle stage). That determines increase of the intraocular pressure with consecutive atrophy of the optic nerve.

The most frequent causes are those associated with retinal hypoxia, such as proliferative diabetic retinopathy, central retinal vein occlusion, branch retinal vein occlusion, central retinal arterial occlusion, ischemic ocular syndrome etc.

Rare causes of neovascular glaucoma are multiple and are due to VEGF synthesis associated with chorioretinal inflammations or degenerations.

We present a case with neovascular glaucoma associated with a rare cause, angioid streaks.[**1**]

## Methods

Case report of patient MC, 48 years old female which presented at emergency room with red and painful left eye. 

The patient relates that for the last 10 years the lefteye visual acuity was decreased but she has not performed any investigation concerning the cause. Left eye best-corrected visual acuity was 1/10. Uncorrected right eye visual acuity was 1. Left eye intraocular pressure 36 mm Hg, right eye intraocular pressure 17 mm Hg.

**Fig. 1:**
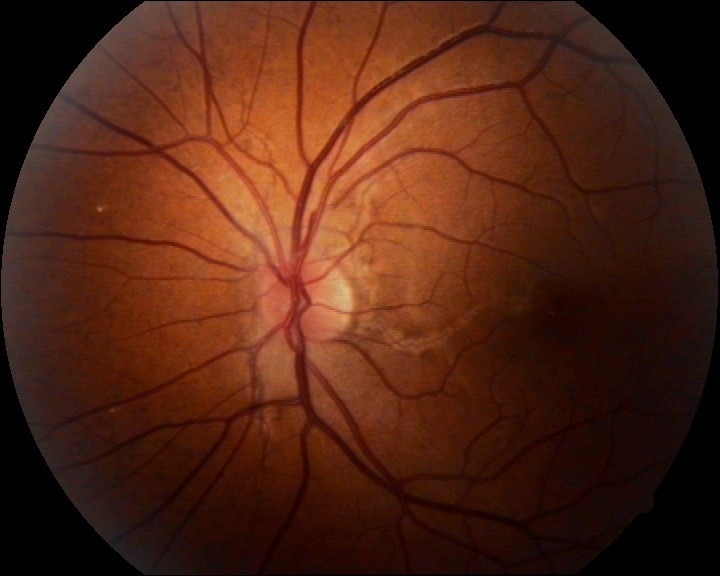
Left eye angioid streaks with foveal invovment and macular edema

Slit lamp examination: left eye conjunctival hyperemia, epitelial corneal edema, rubeosis iridis; right eye was normal. Retinal examination - both eyes angioid streaks, left eye with foveal involvement and macular edema. The examination of the left eye was difficult due to the corneal edema (**Fig. 1**). The ocular and general examination did not provide any other possible cause for neovascular glaucoma.

## Results

The patient was admitted in the hospital. After general (intravenous Manitol and oral Acetazolamid) and topical treatment with fixed combination timolol-brinzolamide, topical steroid and mydriatic the intraocular pressure has decreased (left eye intraocular pressure = 18 mm Hg) and reduction of the corneal edema. Gonioscopy reveals anterior chamber fibrovascular membrane.

**Fig. 2:**
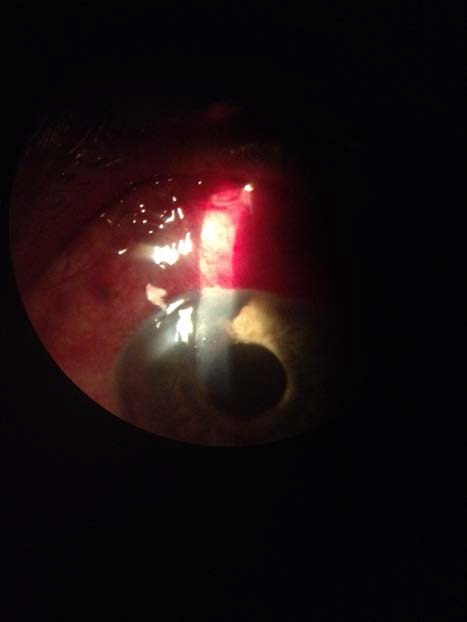
Premature AGA IDM

We performed a vitreal Bevacizumab injection with was followed by resorbtion of the fibrovascular membrane and rubeosis iridis and decrease of the macular edema. 3 weeks after the Bevacizumab injection we performed 5-FU assisted trabeculectomy. (**Fig. 2**). The evolution was favorable with decrease of intraocular pressure without any glaucoma treatment (right eye intraocular pressure = 17 mm Hg). At 6 month follow-up the pressure was stable.

## Discussion

Angioid streaks are small breaks in Bruch's membrane, often with a radial disposition around the optic nerve. They are frequently associated with pseudoxanthoma elasticum, which was not the case for our patient. Sometimes one of the breaks interests the fovea and might be able to determine choroidal neovascularization. [**1**-**3**] Association with neovascular glaucoma is extremely rare because usually angioid streaks are not associated with the degree of retinal ischemia necessary for an important increase of the VEGF synthesis.

The intravitreal injection of Bevacizumab was efficient and determined the resorbtion of the fibrovascular membrane, decrease of intraocular pressure and of the macular edema. There were reports that anti-VEGF injection might determine fibrosis of extramacular angioid streaks.Trabeculectomy was also efficient and maintained its efficacy at the 6 month follow-up.

**Sources of Funding**

None

**Disclosures**

None
